# The effectiveness of mindfulness-based stress reduction (MBSR) for survivors of breast cancer: study protocol for a randomized controlled trial

**DOI:** 10.1186/s13063-016-1335-z

**Published:** 2016-04-22

**Authors:** Jiayan Huang, Lu Shi

**Affiliations:** Key Laboratory of Health Technology Assessment, Ministry of Health (Fudan University), 130, DongAn Road, 200032 Shanghai, China; Department of Public Health Sciences, Clemson University, 525 Edwards Hall, Clemson, SC 29634-0745 USA

**Keywords:** Mindfulness-based stress reduction, Breast cancer survivor, Quality of life, Compliance

## Abstract

**Background:**

After treatment completion, breast cancer (BC) survivors frequently experience residual symptoms of pain, fatigue, high levels of psychological stress, anxiety, depression, fear of recurrence, and metastasis. Post-treatment stress, in particular, can adversely affect health-related quality of life, which, in turn, induces onset or recurrence of chronic diseases. Effective interventions that target these psychological symptoms and their physiological consequences are needed, especially for economically disadvantaged patients. However, in China, few evidence-based intervention strategies have been established among BC survivors. This study will formally adapt, develop, and evaluate an intensive mindfulness-based stress reduction (MBSR) intervention protocol to improve mental health, quality of life, and compliance with medication among Chinese BC survivors.

**Methods:**

A randomized, waitlist-controlled clinical trial will be conducted. Based on our power calculation, 418 BC survivors will be recruited from 10 low-income communities in Shanghai. All subjects will be randomly assigned either to the MBSR program or to a waitlisted usual care regimen that will offer the MBSR program after the completion of the other trial arm (after 6 months follow-up). Our 8-week MBSR intervention program will provide systematic training to promote stress reduction by self-regulating arousal to stress. Assessments will be made at baseline, 4 weeks (in the middle of the first MBSR intervention), 8 weeks (at the end of the first MBSR intervention), 6 months, and 12 months, and will include measures of psychological symptoms (depression, anxiety, and perceived stress), quality of life, and medication adherence. The expected outcome will be the improvement in psychological symptoms, quality of life, and medication compliance in the MBSR intervention group.

**Discussion:**

This study will help develop an affordable, self-care psychological intervention protocol to help Chinese BC survivors improve their quality of life, and could be helpful in further developing affordable disease management plans for patients of other chronic diseases.

**Trial registration:**

ChiCTR-IOR-14005390 (10/27/2014)

## Background

Breast cancer (BC) is a major public health challenge, accounting for 16.8 % of all cancer cases in China among women aged above 18 years [1]. With a 5-year all-stage survival rate of 85 % [1], BC prevalence in China has continued to increase over the past two decades, with a decrease in the age of incidence [2]. Even though the survival rate for stage I or II BC patients has improved, the emotional impact of a cancer diagnosis and the side effects of cancer treatment (e.g., body incompleteness after mastectomy, hair loss after chemotherapy) have elevated the stress levels of many BC survivors [3]. Evidence demonstrates that almost 60 % of BC patients report high levels of anxiety, while 25.6–58 % report living with depression [4, 5]. This prevalence level is much higher than that of the general population, and is even higher than that of patients of other cancer types. Emotional distress adversely affects these women’s quality of life (QOL) and this may persist beyond treatment [6].

After women have completed main treatments (such as chemotherapy and radiotherapy) for BC, they have to face the stage of “watchful waiting”, a stressful period when the physician seems to be “doing nothing”. Therefore, survivors continue to report remaining physical symptoms of pain, fatigue, and sleep dysfunction, high levels of psychological stress, anxiety, depression, fear of recurrence and metastasis, and impaired QOL [7, 8]. This psychological impact adversely affects neuroendocrine stress response systems, which may lead to immunological disturbance [9] and may even contribute to the recurrence or progression of the disease [10]. Further, these mental health challenges may lower the social functioning of the patient or affect her compliance with further treatments (like oral drug therapy) [11]. It is thus no surprise that psychosocial variables are key predictors of both short-term and long-term QOL [12, 13]. Consequently, psychosocial interventions need to be used as an important supportive therapy for minimizing mortality and morbidity among BC survivors. Evidence shows that the post-treatment stage seems to be an ideal period to introduce stress reduction interventions [14]. However, only very limited research and treatment regimens have been developed to minimize this high degree of morbidity during the difficult transitional period of post-treatment survivorship.

In China, to the best of our knowledge, there have been relatively few psychosocial interventions for cancer patients or survivors, with a particular scarcity of effective stress reduction interventions. Wan (2008) [15] analyzed the impact of a psychological intervention on stress by measuring cortisol and interleukin-2 in perioperative BC patients. Li [16] reported the effects of psychological interventions on illness uncertainty and coping styles of BC patients undergoing chemotherapy. Nevertheless, these intervention studies have mainly been designed to decrease stress and improve physical and psychological functioning among BC patients while on treatment or at diagnosis. Furthermore, the psychological intervention programs used in these studies are based on usual care, including health education and language guidance for patients. Since such interventions have not been developed into established protocols, the applicability to psychological stress relief effect is limited, and there is no evidence that the intervention effect is sustainable. These studies have been carried out simply as pilot studies without follow-up trials, and thus their treatment procedures have not been fully developed into policies or protocols. Further, survivors from low-income households may not be able to afford expensive psychosocial therapies or intervention programs in psychiatric hospitals, even if they need the care. Zhou et al. [17] analyzed the average outpatient expenses for depressive patients in Shanghai, showing that the average outpatient expense was 264 RMB per visit in 2005, 71 % higher than in 2002, after adjusting for consumer price index. Thus, it is necessary to develop an affordable, self-care intervention protocol for mental health in BC survivors, especially for economically disadvantaged patients who still need to work to pay for their follow-up treatments and monitoring.

Mindfulness-based stress reduction (MBSR) uses meditation to cultivate conscious awareness (i.e., mindfulness) of one’s experience in a non-judgmental or accepting manner [18]. MBSR includes both didactic and experiential elements that focus on mindfulness-based meditative practices [19]. It is premised on the belief that bringing greater awareness to actual experience in the “here and now” encourages a disengagement from self-related thoughts (e.g., rumination) and emotions (e.g., anxiety) that can have a detrimental effect on well-being [20]. Generally, a MBSR intervention program is 6 or 8 weeks long and includes weekly 2-hour group sessions where participants receive systematic training to promote stress reduction by self-regulating mindfulness practices such as sitting meditation, body scan, and yoga, among others. Participants are also taught informal practices which emphasize bringing mindfulness practice into day-to-day life, and require home practice of 15–30 minutes per day.

MBSR’s effectiveness in reducing stress has been proven in different populations, including patients with chronic pain [21], anxiety [22, 23], and depression [24]. The effectiveness of MBSR in mental health has also been documented for women with early-stage BC [25]. Henderson et al. [26] tested the effectiveness of a MBSR program compared with a nutrition education intervention and usual care in women with newly diagnosed early-stage BC undergoing radiotherapy, and confirmed its efficacy in facilitating psychosocial adjustment in these women. Witek-Janusek et al. [27] showed that an MBSR program was feasible for women recently diagnosed with early stage BC and provided preliminary evidence of its beneficial effects on immune function, QOL, and coping effectiveness. However, as a lifestyle-oriented, non-pharmacological intervention, with its long-term self-sustainable feature [23], MBSR has only been used in China for populations with no clinical indications (like nurses, students) to reduce stress [28].

Many MBSR studies among cancer survivors have at least one of the following limitations: small sample size (less than 100), short follow-up (just after intervention program ends), or a high ratio of Caucasian participants. Moreover, no studies to date have explored MBSR’s possible impact of enhancing patient compliance with treatment guidelines, even though its effect in improving working memory suggests that MBSR might help patients to better maintain medication and hospital follow-up schedules [29]. In this proposed study, we plan to use MBSR to improve the mental health and treatment compliance among BC survivors. Our proposed MBSR intervention will fill the research gap by exploring its impact on adjuvant therapy compliance in addition to mental health outcomes.

## Study objectives

### Overall goal of the project

This proposed study attempts to improve BC survivors’ mental health and QOL, especially those in low-income families to reduce inequity in mental health care.

This project has two phases: a pilot study and the main study.

### Objectives of the pilot study

- Modify the MBSR intervention program to suit the Chinese context.
- Revise the measuring instruments to suit the Chinese context and the intended subjects (women with low income).
- Assess the feasibility of the proposed procedures.

### Objectives of the main study

- Evaluate the effect of MBSR intervention program in BC survivors.
- Develop a special affordable, non-medical, self-care psychosocial intervention protocol for Chinese BC survivors.
- Develop policy recommendations for mental health among BC survivors.

## Theoretical model and hypotheses

### Theoretical model

**Fig. 1:**
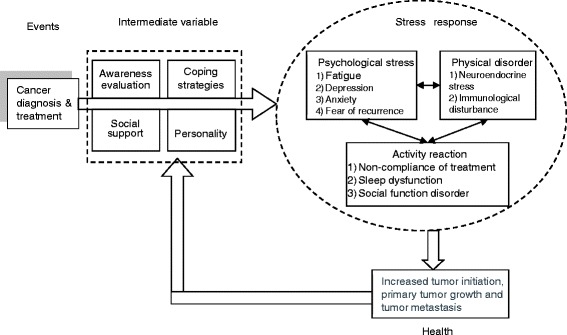
A theoretical model of breast cancer survivors’ psychosomatic response mechanism

The psychosomatic response mechanism in BC survivors is graphically presented in Fig. 1. As discussed above, cancer diagnosis and treatment would adversely affect BC survivors’ stress reaction, but the stress level may be influenced by intermediate variables (like survivors’ awareness to disease, coping strategy, social support, and personality). For the various types and intensities of these intermediate variables, different BC survivors would have different psychosomatic responses. BC survivors frequently experience high levels of psychological stress, which is often accompanied by dysregulated neuroendocrine function and immunological disturbance. These impairments are associated with increased tumor initiation, primary tumor growth, and tumor metastasis, which is the primary cause of death from BC [30–32].

**Fig. 2:**
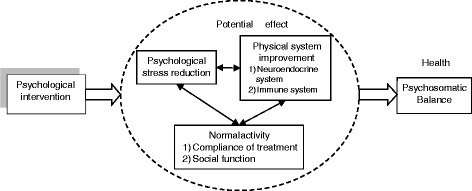
A model of psychological intervention’s mechanism

MBSR is a mind-body intervention that shows promise for the management of stress associated with cancer. We hope to show that MBSR reduces neuroendocrine stress activation and improves the immune function of BC survivors after the vulnerable periods of cancer diagnosis and treatment [33, 34]. This would in turn further contribute to improvement of QOL among BC survivors, which would promote psychosomatic balance (Fig. 2).

### Primary hypotheses

1. Participants in MBSR would show improvements in mental health, QOL, and medication treatment compliance, relative to control subjects. We predict that MBSR would help reduce psychological distress and increase patients’ ability to monitor negative cognitions and thereby decrease stress.
2. The levels of mindfulness would increase over the course of the 8-week MBSR program, serving as a mediator to positive changes in mental health, QOL, and medication treatment compliance.

### Secondary hypotheses

1. MBSR would be an intervention modal with a long-term effect. We predict that at 1-year follow-up, the MBSR group would still show improvements in mental health, QOL, and medication treatment compliance, compared with its baseline levels.

## Methods/Design

In this study, a randomized, waitlist controlled trial design will be used. The clinical trial registry number is ChiCTR-IOR-14005390.

### Setting and participants

Shanghai will be selected as the site of the study because it has been characterized by a high incidence of BC in China in recent years [35]. Participants in low-income households will be recruited from about 10 different communities from two of Shanghai’s poorer districts: Yangpu (characterized by an inner-city urban poor population of Shanghai origin) and Minhang (characterized by a migrant worker population).

Inclusion criteria are (1) female, (2) with a history of stage 0, I, II, or III BC, (3) within 2 years from the date of BC diagnosis, (4) currently under at least one adjuvant therapy.

Exclusion criteria are (1) current treatment for recurrent BC, (2) a history of schizophrenia or schizo-affective disorder [24], and (3) current alcohol or drug abuse.

### Intervention

#### MBSR intervention

Subjects assigned to the MBSR intervention group will receive weekly 2-hour sessions conducted by a psychologist certified and trained in MBSR. Class sizes may range from 20 to 30, with 8–10 groups in total. All sessions will be standardized and will follow the training manual to be developed to maintain consistency in the program. A trained psychologist will deliver the intervention. In addition, an observer will monitor the weekly sessions for inter-group consistency by recording the timing and other aspects of the intervention, and the quality of each session will be assessed in a qualitative post-observation report. Subjects will receive a training manual to support home practice of various forms of meditations (sitting meditation, body scan, and walking meditation) and gentle yoga. The training manual included weekly objectives, exercises, and program content related to the content identified below. In addition, the manual will include a daily diary for recording practice activities at home.

Based on Kabat-Zinn et al.’s MBSR program [22], this intervention will be an 8-week program adapted for the Chinese context, particularly for women in poorer socioeconomic situations. The objective of the intervention is to train participants to reduce their level of stress by self-regulating arousal to stressful circumstances or symptoms. At the beginning of each week, time will be provided for participants to discuss their experience, thus enhancing group interactions. More specifically, during week 1, participants will be given an overview of the intervention to establish a learning contract and to learn initial meditation and body scan procedures. In week 2, participants will learn visualization and will be introduced to sitting meditation with awareness of breathing as the primary object of attention. During weeks 3 and 4, participants will gain an understanding of one’s reaction to a pleasant event, body scan with response to stress, and introduction of yoga postures. Week 5 will instead provide participants with an understanding of their reaction to unpleasant rather than pleasant events. During weeks 6 and 7, participants will expand their field of awareness to allow for modification of stress-inducing patterns, and continue to monitor their awareness to allow these through techniques such as mountain meditation and/or lake meditation; awareness will be expanded to include objects such as bodily sensations, sounds, thoughts, and feelings. Finally, in week 8, participants will be encouraged to internalize practice sessions and develop a pattern for themselves. Participants will be told that week 8 is the first week of being on their own and to develop a lifetime program with the emphasis that meditation is a means to access wellness.

This modified intervention provides for management of specific emotional/psychological symptoms (anxiety, depression, and fear of recurrence) and physical symptoms, such as pain and sleeplessness. Through the use of meditation practices (sitting meditation, body scan, and walking meditation) and yoga, subjects are taught to increase awareness of their thoughts and feelings and to observe their emotional and physical responses during stressful situations. Through the process of mindfulness attention, subjects will take an active role in regulating their stress and managing symptoms and emotions, thus enabling them to cope better with the distress of having cancer. Administratively, the intervention will have three specific components: (1) educational material related to relaxation, meditation, and mind-body connection; (2) practice of meditation in group meetings and homework assignments; and (3) discussion among group members about barriers to practicing meditation, application of mindfulness in daily situations, and group support.

Throughout the 8-week MBSR program, all subjects will be requested to formally meditate (sitting, walking, and body scan exercises) 6 days per week. They will also be asked to informally practice 15–30 min per day, and this time will increase per week as participants become more experienced. Daily practice will be recorded in a diary each day. The total number of minutes/hours practiced over the 8-week program will also be recorded for analysis.

#### Usual care regimen

Subjects randomized to the waitlisted control group will receive no formal intervention, but will continue to have standard post-treatment clinic visits according to their treatment plans. Additionally, to avoid possible overlap (contamination) with components of the MBSR program, the investigators will specifically ask subjects in the waitlisted control group not to start mindfulness-based practice during the study period.

MBSR intervention will be offered to each usual care subject after 6 months follow-up. They will be provided with a brief orientation to MBSR (BC), a manual on the program, and a 2-month practice class after post-assessment.

### Outcomes

#### Expected outcomes

1) Improvement in mental health, QOL, and meditation adherence in the MBSR intervention group, which is generalizable to East Asia’s growing population of BC survivors. As our 2010 commentary in *Science* states [36], the age of BC incidence among East Asian women tends to be substantially younger than that of Caucasian women. Therefore, good mental health among this population of BC survivors also means a labor productivity gain for China’s shrinking labor force. 2) A lifestyle-oriented, non-pharmacological psychological intervention protocol to help BC survivors to self-control their stress, which will become easier to access among low-income communities thanks to the training program in our study.

The following instruments would be used to measure the outcome and the possible mediators.

#### Practice quantity

A meditation calendar will be provided to participants to document the daily number of minutes spent in formal meditation practice, and the participants are expected to document this figure at the end of each day during the intervention. Calendars will be collected at the end of the intervention, following the practice of mindfulness intervention in smoking abstinence [37].

#### Practice quality

The 7-item mindfulness practice quality questionnaire [38], which measures the dimension of perseverance and the dimension of receptivity, will be conducted at each session following formal meditation practice.

#### Psychological assessment

Several psychometric scales will be used.

1. Self-rating Depression Scale (SDS) [39]. SDS is a 20-item instrument with a 4-point Likert scale. Higher scores indicate higher depression level.
2. Self-rating Anxiety Scale (SAS) [40], a 20-item instrument with a 4-point Likert scale. Higher scores indicate higher anxiety level.
3. Mindful attention awareness scale (MAAS) [41], which includes a 15-item instrument designed to assess the frequency with which an individual is openly attentive to and aware of present events and experiences. The scale assesses mindfulness of both internal states (e.g., emotions) and overt behaviors (e.g., attention to tasks and social interactions) on a 6-point Likert scale. Higher scores indicate higher mindfulness.
4. Quality of Life Index – cancer version III [42], which evaluates QOL in terms of an individual’s satisfaction or dissatisfaction with aspects of life that are important to him/her. The Quality of Life Index measures life satisfaction in four domains: health and functioning, socioeconomic, psychological/spiritual, and family. It consists of two parts with 34 items, using a 6-point Likert scale. Part I measures satisfaction within each identified domain, whereas part II measures the perceived importance of each item. Satisfaction scores are weighted by importance.

#### Compliance level with medication treatment

1. Morisky Medication Adherence Scale, 8-item version (MMAS) [43], which will be modified to assess subject’s medication adherence. It has 8 items and scores range from 0 to 8. Scores of <6 indicate low, scores of 6–7 indicate medium, and a score of 8 indicates high adherence.

#### Compliance level with intervention

Questionnaire items will be developed to record subjects’ MBSR practice at home, including practice types, number of minutes, and so on.

#### Demographic and health history form

Demographic information will be collected, including age, marital status, education, and employment status. Medical records will be reviewed to obtain information regarding age at BC diagnosis, cancer pathology, past and current cancer treatment, and patient’s compliance with treatment. At each assessment, a health history form will be completed by the participants, including questions related to presence of other medical conditions or diseases, current use of prescription and non-prescription medications and supplements, and occurrence of recent illness.

### Sample size

Based upon an MBSR study among BC survivors where the intervention group had significantly better aggregated physical health outcome as measured by SF-36 (50.3 among MBSR subjects vs. 46.9 among the control group) [44], we used STATA 12.0’s power calculator to find that our two-group, three-follow-up design needs 195 participants for each of the two groups, i.e., 390 subjects in total. Assuming an attrition rate of 10 % (attrition rate here means the percent of people who drop out from the study due to moving away, death, etc.), we concluded that we need to recruit a sample of 429 BC survivors at baseline.

### Randomization

#### Sequence generation

Two investigators in our team will be in charge of implementing the random allocation sequence after they get the whole list of registered patients. All these registered patients will be sorted according to the order of Chinese name, then given number “1” and “2” in sequence. The patients with “1” will be assigned to the immediate intervention group and those with “2” to a waitlist control group.

#### Allocation concealment

We will not use “treatment” or “control” group to mention the allocation. All the subjects will be told that they will be arranged into two groups to attend the MBSR session separately due to resource limitations. Four investigators will call the subjects to let them know the group to which they belong, either the immediate group or the waitlist group.

#### Implementation

Following completion of their hospital treatment, BC survivors return home. As part of the newly launched cancer registry system, each community health service center (CHSC) in Shanghai collects BC survivors’ detail information in order to ensure follow-up and to analyze mortality and morbidity rates. Based on the patient lists from CHSC, we will contact these BC survivors directly to recruit potential participants. All participants who are willing to attend this trial will be requested to submit a signed informed consent. After that, all participants who provide an informed consent will then complete a baseline questionnaire for psychometric assessment (Time 0), with the help from investigators. Information about demographic background and health status will also be collected and later verified by a review of hospital charts.

The immediate intervention group will start the program within 2 weeks of randomization. Two certified MBSR psychotherapists will be hired to implement a standard MBSR intervention. Members of the research team will attend each MBSR session to ensure that intervention standards are met.

All subjects will be reassessed after the intervention group completes half of the program (4 weeks into the intervention, Time 1), 8 weeks later (Time 2), and at 6 (Time 3) and 12 months (Time 4) from the beginning of the intervention. Participants in the control group will be given the opportunity to complete the MBSR program after 6 months follow-up. Eight investigators will be in charge of these follow-ups by telephone.

### Statistical methods

1. We will compare the outcome variables (SDS, SAS, MAAS, Quality of Life Index, and MMAS) between the two groups using the χ^2^ test for categorical variables and Student t or Wilcoxon tests for continuous variables. Analysis of covariance (ANCOVA) will be conducted to assess whether MBSR is related to changes between baseline and after 8 weeks of intervention in relation to psychological status, QOL, and compliance behaviors.
2. We will use a structural equation model [45] to examine the intervening role of mindfulness enhancement on improving mental health, QOL, and compliance with medical treatment. Mediation analysis, as implemented in MPlus 7.11, will be used to analyze the causal pathway from MBSR intervention to health outcomes through mindfulness enhancement.
3. We will use a random-effects repeated measures analysis [46] to examine the impact of MBSR intervention on health outcomes, compliance behavior, adjusting for confounding variables (age, education, household income, occupation, previous cancer history, and household registration status: Shanghai vs. non-Shanghai). The repeated measure analysis approach accounts for the same individual’s different outcome measures across different points in time without assuming either linear or curvilinear growth pattern. Cronbach alpha’s will be computed to determine the internal consistency of our outcome measures. Using this approach (as implemented by the PROC MIXED and PROC GLIMMIX modules in SAS 9.0), we have published an article analyzing a famous randomized controlled trial of lung cancer screening, which shows a behavioral differential between the two trial arms [47].

The relationship between methods used for each hypothesis is shown in Table 1.

### Pilot study

A small-scale pilot study will be carried out in one low-income community in Shanghai. We will recruit 20 BC survivors according to our inclusion criteria and baseline assessment will be done before intervention. After that, the MBSR intervention program will be offered to each subject as a group session. At the end of 8-week intervention, all subjects will be reassessed. We will also collect subjects’ feedback regarding the intervention program. On the basis of the experience and feedback:

1. We will modify the content of MBSR, if needed, in order to make it more suitable for Chinese BC survivors.
2. We will revise our assessment instruments according to subjects’ feedback.

### Procedures to ensure compliance with ethical standards

- All participants will be asked to provide written informed consent and will be assured of anonymity and confidentiality. In this letter, apart from the brief introduction of this project, subjects will be informed about the detailed rules on the protection of their information, as follows: (1) each investigator in this project will be requested to sign a confidentiality agreement to ensure that they will not disclose information of subjects; (2) research data will be collected with absolute anonymity and confidentiality; and (3) only registered investigators can access the data and outcomes.
- We have research ethics approval from Ethics Committee of Fudan University School of Public Health, and the IRB Approval Number is 2014-TYSQ-09-1.

## Discussion

The largest threat to our study’s internal validity is the possible “contamination effect” (or spillover effect) between the two trial arms. As our participants will be recruited from 10 low-income communities from two of Shanghai’s poorer districts, it is possible that some of these participants know each other and may hold discussions regarding the MBSR treatment. The contamination effect could compromise measurable differences between the two trial arms and bias the intervention result toward the null hypothesis. However, as MBSR takes a substantial amount of exercise to achieve measurable results in mental health, it is unlikely that contamination alone could diminish the difference between those who attend the class and those who only hear about the practice. Plus, we will communicate to the waitlist control group in written materials to make sure that they start MBSR practice only when they receive the intervention.

**Table 1:** Summary of methods used for each hypothesis

Objective (main study)	Hypothesis	Method
Objective 1	Primary Hypothesis 1: Improvement in mental health, quality of life, and medication treatment compliance in mindfulness-based stress reduction (MBSR) group	Method 1: Bivariate analyses and ANCOVA
	Primary Hypothesis 2: Relation between MBSR and improvement	Method 2: Mediation analysis in structural equation modeling
Objective 2	Secondary Hypothesis: Long-term improvement of MBSR	Method 3: Repeated measures analysis
Objectives 3 and 4	N.A.	Expert consultation

There is also the possible threat to internal validity through high attrition among participants. Attending an 8-week course is not a trivial matter for young working women, and thus it is possible that those who do not experience an immediate gain in mental health are more likely to quit. This possible pattern of selective attrition could lead to overestimation of intervention impact, as most of those who stay until the end of the intervention are more likely to experience MBSR benefits. We believe, however, that our financial incentive for participants should be sufficient for most BC survivors to complete the course, so long as we appropriately program the timing of our financial incentive (e.g., payment will be given out at each session, instead of being given out in a lump sum at the beginning).

## Trial status

At the time of writing of this manuscript the trial had not yet begun. We are currently applying for financial support.

## Abbreviations

MBSR: Mindfulness-based stress reduction, an intervention to reduce the stress by mindfulness
BC: Breast cancer
QOL: Quality of life
